# The Conserved Residue Arg46 in the N-Terminal Heptad Repeat Domain of HIV-1 gp41 Is Critical for Viral Fusion and Entry

**DOI:** 10.1371/journal.pone.0044874

**Published:** 2012-09-07

**Authors:** Xiaoyi Wang, Weiliang Xiong, Xiaochu Ma, Meili Wei, Yanxia Chen, Lu Lu, Asim K. Debnath, Shibo Jiang, Chungen Pan

**Affiliations:** 1 Key Laboratory of Tropical Disease Control of MOE, Department of Biochemistry and The Institute of Human Virology, Zhongshan School of Medicine, Sun Yat-Sen University, Guangzhou, China; 2 Key Laboratory of Medical Molecular Virology of Ministries of Education and Health, Shanghai Medical College and Institute of Medical Microbiology, Fudan University, Shanghai, China; 3 Lindsley F. Kimball Research Institute, New York Blood Center, New York, New York, United States of America; University Hospital Zurich, Switzerland

## Abstract

During the process of HIV-1 fusion with the target cell, the N-terminal heptad repeat (NHR) of gp41 interacts with the C-terminal heptad repeat (CHR) to form fusogenic six-helix bundle (6-HB) core. We previously identified a crucial residue for 6-HB formation and virus entry - Lys63 (K63) in the C-terminal region of NHR (aa 54–70), which forms a hydrophobic cavity. It can form an important salt bridge with Asp121 (D121) in gp41 CHR. Here, we found another important conserved residue for virus fusion and entry, Arg46 (R46), in the N-terminal region of NHR (aa 35–53), which forms a hydrogen bond with a polar residue, Asn43 (N43), in NHR, as a part of the hydrogen-bond network. R46 can also form a salt bridge with a negatively charged residue, Glu137 (E137), in gp41 CHR. Substitution of R46 with the hydrophobic residue Ala (R46A) or the negatively charged residue Glu (R46E) resulted in disruption of the hydrogen bond network, breakage of the salt bridge and reduction of 6-HB’s stability, leading to impairment of viral fusion and decreased inhibition of N36, an NHR peptide. Similarly, CHR peptide C34 with substitution of E137 for Ala (E137A) or Arg (E137R) also exhibited reduced inhibitory activity against HIV-1 infection and HIV-1-mediated cell-to-cell fusion. These results suggest that the positively charged residue R46 and its hydrogen bond network, together with the salt bridge between R46 and E137, are important for viral fusion and entry and may therefore serve as a target for designing novel HIV fusion/entry inhibitors.

## Introduction

The fusion of human immunodeficiency virus 1 (HIV-1) and its target cell is mediated by the envelope glycoprotein consisting of surface subunit gp120 and transmembrane subunit gp41 which are associated with non-covalent interactions [1]. To initiate infection, the gp120 binds to its receptor CD4 on the surface of the target cell and then to coreceptors (CCR5 or CXCR4), events which trigger a cascade of conformational changes of gp41, facilitating the fusion between membranes of HIV and its target cell [2], [3], [4].

**Figure 1:**
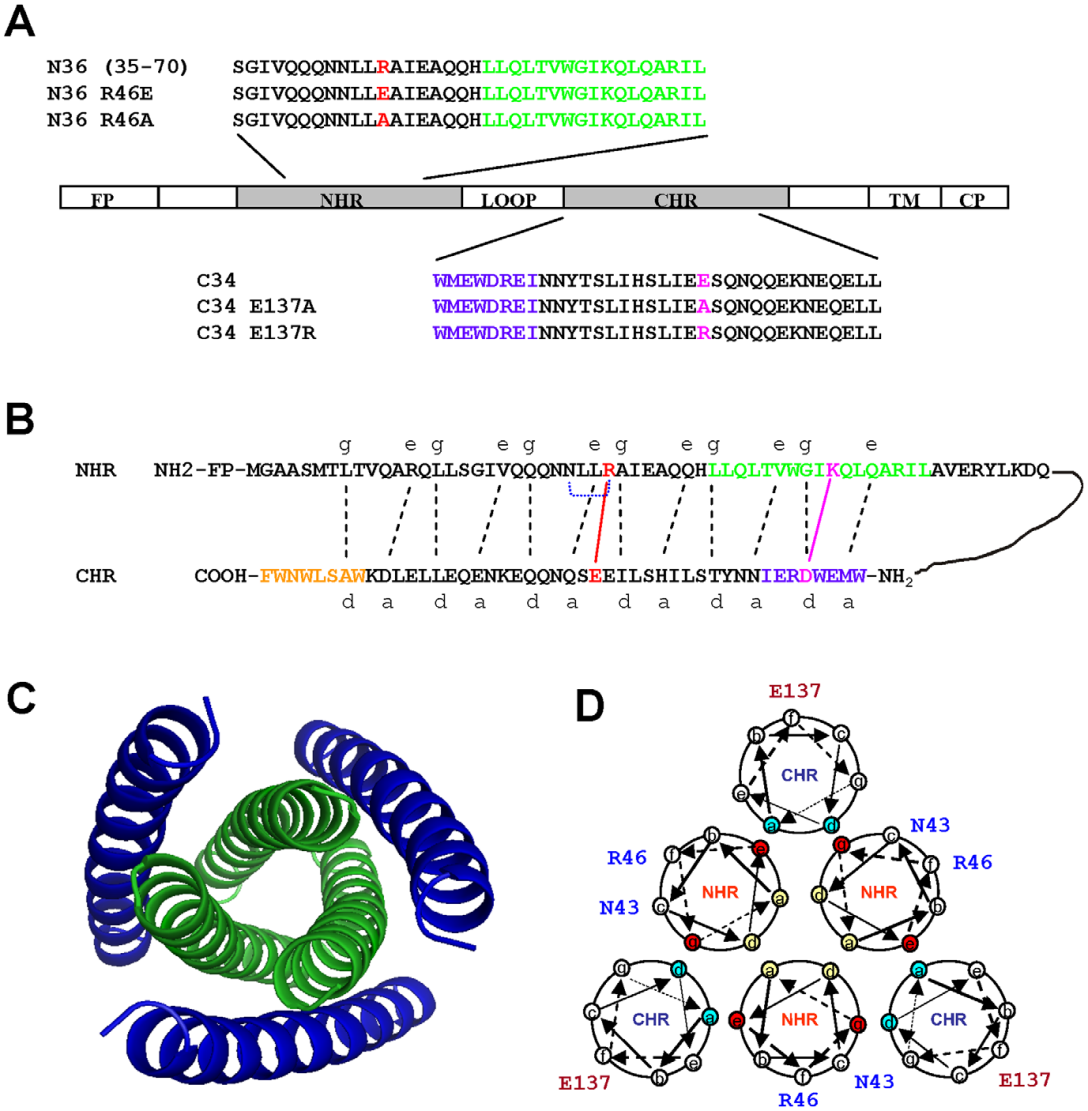
Schematic representations of the HIV-1 gp41 molecule, the core structure and the NHR/CHR interactions. (**A**) The functional domains in the gp41 molecule and the sequences of the NHR peptide N36 and the CHR peptide C34, as well as their mutants. (**B**) Interactions between the amino acid residues in the gp41 NHR and CHR. The black dashed lines between the NHR and CHR domains indicate the interaction between the residues located at the *e, g* and the *a, d* positions in the NHR and CHR, respectively. The red and pink solid lines represent the ionic interactions between R46 and E137, as well as K63 and D121, respectively, while the blue dotted line denotes the hydrogen bond between R46 and N43. Pocket-forming domain (PFD) in the NHR and pocket-binding domain (PBD) in CHR, as well as lipid-binding domain (LBD) in the MPER, are highlighted in green, violet and orange, respectively. (**C**) X-ray crystal structure of the HIV-1 gp41 6-HB core formed by N36 and C34 (adapted from [5]). NHR is colored in green, and CHR is colored in blue. (**D**) Model of the gp41 6-HB showing the locations of R46 and N43 in the N-helix wheel and E137 in the C-helix wheel. The residues located at the *a, d* positions (yellow) in one of the N-helices interact with those at the *d, a* positions (yellow) in another N-helix, respectively, resulting in formation of the NHR-trimer. The residues located at the *e, g* positions (red) in one of the N-helices associate with those at the *a, d* positions in one of the C-helices, respectively, leading to the formation of 6-HB.

The HIV-1 gp41 consists of three major functional domains, including the fusion peptide (FP), the N-terminal heptad repeat (NHR), and the C-terminal heptad repeat (CHR). The peptides derived from the NHR and CHR, e.g., N36 and C34, exhibited potent anti-HIV-1 activity (**Figure 1A**) [5], [6]. Previous studies have revealed that the conformation of gp120/gp41 complex finally changes from native state to a hairpin state through a pre-hairpin fusion intermediate (PFI) [7]–[9]. In the fusion state, the residues at the ***a*** and ***d*** positions of one NHR domain interact with those at the ***d*** and ***a*** positions of another NHR domain to form N-helix trimer, while the residues at the ***e*** and ***g*** positions of one NHR domain interact with those at the ***a*** and ***d*** positions of the CHR domain to form a six-helix bundle (6-HB) core (**Figure 1B and C**), in which three N-terminal heptad repeats (NHR) form an interior, parallel coiled-coil trimer with three C-terminal heptad repeats (CHR) inserting into its highly conserved, hydrophobic cavity on the surface [5] (**Figure 1D**).

Considerable evidence indicates that hydrophobic interaction in the deep hydrophobic pocket is critical for the stabilization of six-helix bundle and virus infectivity [10], [11], [12]. Some mutations in the crucial conserved residues of CHR and NHR involving inter-helical interactions within the cavity can destabilize the structure of gp41 and thus inhibit membrane fusion and reduce the infectivity of the virus [13]–[18]. Furthermore, some peptides, such as Enfuvirtide [19] and Sifuvirtide [20], both of which mimic the structure of CHR, proved to be effective in inhibiting viral entry and now serve as a novel class of HIV fusion inhibitors.

Apart from hydrophobic interaction, the inter- and intra-helical hydrogen bonds, or salt bridges, distributed along the hydrophobic contacts, especially in the middle of the 6HB, are presumed to be highly related to the stability of 6-HB and virus entry [21], [22]. Based on the trimer-of-hairpins structure of gp41, it has been found that two planar patches of conserved polar residues in both the NHR and CHR, including Gln-40, Gln-41, and Asn-42 of the N-terminal helix, as well as Gln-141 and Gln-142 of the C-terminal helix, can form a network of hydrogen bonds in or near the “glutamine layer” within the six-helix bundle [21]–[25]. Although the functional role of this hydrogen-bond network is poorly understood, available data have demonstrated its correlation with 6-HB stability. Certain substitutions in this layer could change the core stability of gp41 [14], [17], [22], [26] and affect fusogenicity [13]. In addition, a possible T20-resistance mechanism involving the double mutation E137K/N43D has been shown to be largely related to this hydrogen bond formation [27]. However, the effect of hydrogen bonds on viral infection remains unclear.

In the present study, we found that R46, a positively charged residue in the N-terminal region of the gp41 NHR domain, plays an important role in virus fusion and entry. It could bind with N43 in the NHR through a hydrogen bond, which is consistent with previous report [28], as a part of an intricate hydrogen-bond network, and interact with the negatively charged residue E137 in the gp41 CHR domain to form a salt bridge. Substitution of R46 with Ala or Glu resulted in disruption of the hydrogen-bond network and breakage of the salt bridge, as well as reduced stability of 6-HB and decreased effectiveness of viral fusion and entry. Moreover, non-conserved mutations of R46 led to reduced inhibitory activity of the NHR peptide N36, suggesting that R46 and the hydrogen-bond network are important for viral fusion/entry and may serve as a target for development of novel HIV fusion/entry inhibitors.

## Results

### The positively charged residue R46 in the HIV-1 gp41 NHR may participate in formation of a salt bridge with E137 in the CHR and a hydrogen bond with N43 in the NHR of gp41

**Figure 2:**
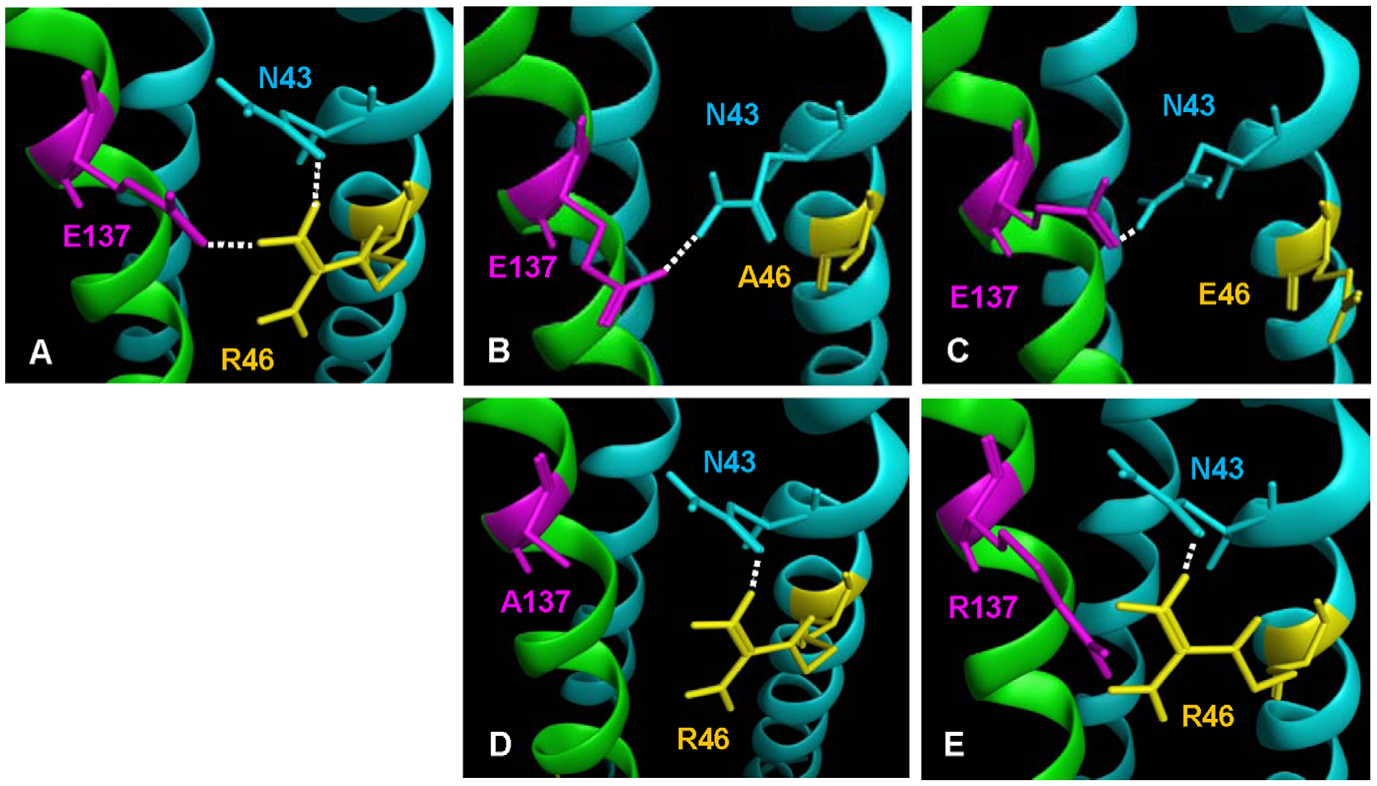
Analysis of putative interactions of R46 in the gp41 NHR with the residues in the 6-HB formed between C34 and N36 or their mutants. (**A**) The 6-HB formed between C34 and N36; (**B**) The 6-HB formed between C34 and N36 R46A; (**C**) The 6-HB formed between C34 and R46E; (**D**) The 6-HB formed between C34 E137A and N36; and (**E**) The 6-HB formed between C34 E137R and N36. The putative interactions via hydrogen bond or salt bridge were predicted by using the MOE program. NHRs are colored in blue; CHR is colored in green.

To analyze the role of key residues in the stability of the N-helix trimer and 6-HB of the HIV-1 gp41, we used Molecular Operating Environment (MOE) program [29] to predict the possible interactions between residues in the gp41 NHR and/or CHR. As shown in **Table 1**, nine pairs of residues in the NHR, such as N43-R46, formed intra-helical hydrogen bonds with energy of more than 0.2****Kcal/mol (**Table 1**). These hydrogen bonds may be important for stabilization of the N-helix and N-helical trimer. Eight amino acid residues in the NHR interacted with those in the CHR to form inter-helical hydrogen bonds with energy of more than 0.2****Kcal/mol, while two salt bridges between the NHR and CHR of gp41 were observed. The first salt bridge between K63 and D121 located in the deep hydrophobic pocket was reported to be critical for the stability of the gp41 6-HB [30]. The second salt bridge is formed between R46 in the N-terminal portion of the NHR and E137 in the CHR (**Figure 1B** **and** **Figure 2** **,** **Table 1**).

**Table 1:** Using the MOE program to predict hydrogen bonds and salt bridges in 6-HB formed by C34 and N36, or N36’s mutants, or by N36 and C34, or C34’s mutants.

6-HBformed by	Energy(Kcal/mol)				
	N36& C34	N36 R46A& C34	N36 R46E& C34	N36 & C34E137A	N36 &C34 E137R
Hydrogen bond or salt bridge in NHR					
I37-Q41	−2.2	−2.2	−2.1	−2.2	−2.2
Q40-Q41	−1.5	−1.4	−1.9	−1.5	−1.5
N43-R46	−0.4	0	0	−0.5	−0.4
I48-Q51	−2	−1.3	−1.3	−2	−2
Q51-Q52	−2.3	−2.4	−2.4	−2.2	−2.3
L54-T58	−1.7	−2	−2.1	0	0
L55-T58	0	−1.7	−1.4	−1.3	−1.7
R68-I69	−1	0	0	−1	−1
Q64-R68	−1.1	−1.1	−2.3	−1.1	−1.1
Hydrogen bond or salt bridge between NHR and CHR					
G36-N145	−0.5	−0.5	−0.4	−0.5	−0.5
G36-E148	0	−0.4	−0.3	0	0
V38-Q142	−0.7	−0.7	−0.8	−0.8	−0.7
Q40-S138	−1.1	−1.9	−1.4	−1.1	−1.1
N42-E146	−2.1	−1.7	−1.6	−1.8	−2.1
N43-E137	0	−0.8	−0.4	0	0
R46-E137	−0.9	0	0	0	0
E49-Q139	−0.8	−0.7	−0.6	−0.6	−0.7
H53-Y127	−0.6	−0.6	0	−0.6	−0.6
Q56-T128	−2	−0.3	−1.6	−3.4	−3.4
L57-W120	−0.6	−0.9	−0.8	−0.6	−0.6
K63-D121	−0.4	−0.6	−0.5	−0.3	−0.4
Total energy between NHR and CHR					
	−9.7	−9.1	−8.4	−9.7	−10.1

* “0” indicates the absence of hydrogen bond or salt bridge between the two corresponding residues.

To investigate the potential role of the newly identified salt bridge between R46 and E137, we replaced R46 with Ala (R46A) or Glu (R46E), respectively, in the 6-HB. Remarkably, these mutations resulted in breakage of the R46-E137 salt bridge and the hydrogen bonds R46-N43 and R68-I69. However, some new hydrogen bonds between the NHR and CHR, such as G36-E148, L55-T58 and N43-E137, were generated (**Figure 2** and **Table 1**). After energy minimization of the structure by the MOE program, the summed energy between NHR and CHR dramatically decreased from 9.7 Kcal/mol (C34/N36) to 9.1 Kcal/mol (C34/N36 R46A) or 8.4 Kcal/mol (C34/N36 R46E), respectively. These results suggest that substitution of R46 with oppositely charged residues, such as Glu, has more profound effect on the stability of 6-HB than the residues without negative charge.

Although substitution of E137 with Ala (E137A) or Arg (E137R) in the CHR led to the breakage of the R46-E137 salt bridge between NHR and CHR in the 6-HB, it had no significant effect on the energy of the R46-N43 hydrogen bond in NHR. Compared with the wild-type 6-HB, the summed energy between NHR and CHR in the mutant 6-HBs formed by N36/C34 E137A or N36/C34 E137R revealed no significant changes (**Table 1**), indicating that mutations of E137 in CHR have less effect on the stability of the HIV-1 gp41 6-HB than those of R46 in the NHR.

### Non-conservative Substitutions of R46 Attenuated the Stability of 6-HB, whereas Mutation of E137 Only Slightly Changed the Secondary Structure of 6-HB

**Figure 3:**
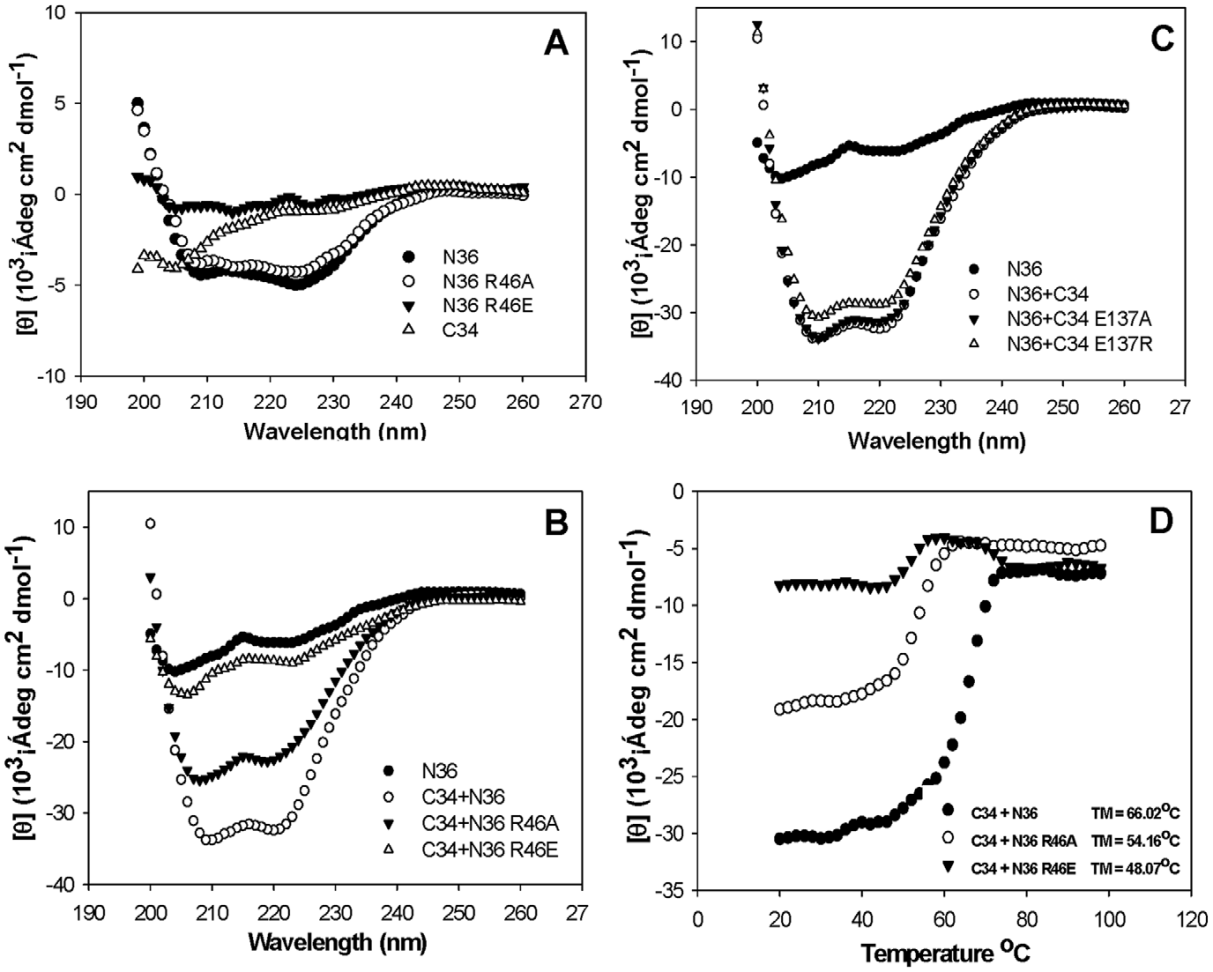
CD spectrographic analysis of the complexes formed between N36 and C34 or their mutants. (**A**) The secondary structures of N36 and its mutants; (**B**) The secondary structures of the complexes formed by C34 and N36 or N36’s mutants; (**C**) The secondary structures of the complexes formed by N36 and C34 or C34’s mutants; and (**D**) The stability of complexes formed by C34 with N36 and its mutants, as measured by thermal denaturation analysis.

To investigate the mechanism of non-conservative substitution of R46 or E137 in determining viral infectivity, we used CD spectroscopy to analyze the interaction between C peptides and N peptides. In CD spectra, classical α-helix secondary structure represents the characteristic double minima at 208 and 222 nm. As shown in **Figure 3A and 3B**, the equimolar mixture of N36 and C34, as the model of fusion-active gp41 core, showed 98% α-helix in its conformation identified by the negative peak in the far UV region and its dramatically increased molar ellipticity (θ) at 220 nm. We observed that the mutation of R46 with Ala (N36 R46A) or Glu (N36 R46E) resulted in the decreased α-helicity of N-helix (**Figure 3A**). This result suggests that these mutations may affect the H-bond between N46 and N43, resulting in disruption of the intricate hydrogen-bond network in the gp41 NHR.

The complex formed by C34 and N36 R46A or N36 R46E indicated a significant decrease in α-helicity, i.e., from 98% (C34/N36) to 79% (C34/N36 R46A) or 26% (C34/R46E), respectively (**Figure 3B**). While the CD spectra of N36 with C34 E137A or C34 E137R do not significantly differ from the mixture of N36 and C34, the α-helicity only changed to 96% (N36/C34 E137A) or 89% (N36/C34 E137R) (**Figure 3C**).

Since N36 mutants have more effect on α-helicity, we used thermal denaturation analysis to investigate the stability of complexes formed by C34 and N36 and their mutants. In **Figure 3D**, C34 and N36 mutant complexes indicated a dramatic decrease in Tm value compared with wild-type 6-HB. This result indicates that non-conservative substitution of R46 can destabilize 6-HB. Furthermore, the Tm value of C34-N36 R46E was significantly lower than that of C34-N36 R46A, suggesting that the substitution of R46 with the negatively charged residue Glu impairs the stability of 6-HB with more potency.

### Non-conservative Substitutions of R46 Affect the Conformation of the 6-HB Bundle

**Figure 4:**
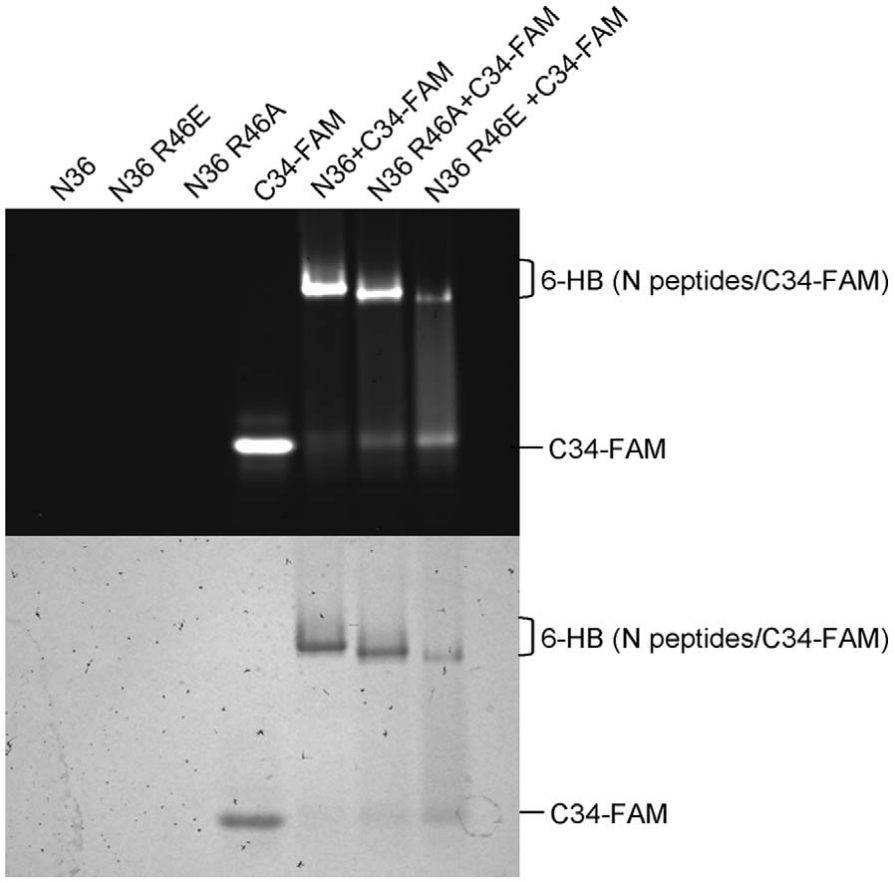
Interaction of C34 with N36 and its mutants to form 6-HB, as determined by FN-PAGE. C34 was labeled with FAM (carboxyfluorescein, Molecular Probes, Eugene, OR), which could be viewed under UV light.

To further study the function of R46, we used fluorescence native PAGE to detect 6-HB formed by carboxyfluorescein (FAM)-labeled C34 (C34-FAM) and N36 bearing non-conserved substitutions. Molecular weights, net charges and different shapes of proteins are determinants of the migration rate of the proteins in electric field. Mutation of one amino acid exerts only slight effect on net charges and molecular weights. Thus, corresponding bands with different migrating rates, if they exist, are mainly caused by the different configurations of 6-HBs which result from the non-conservative substitution of R46. The results showed that N36 and its mimics displayed no band in FN-PAGE because of the net positive charge they contain, whereas C34-FAM showed a distinct band (**Figure 4**). A clear band could be observed when wild-type N36 was mixed with C34, corresponding to the 6-HB. The bands of mutant N36/C34-FAM complexes are specific, but were revealed at lower position than those of the N36/C34-FAM complex. Obviously, the corresponding bands of 6-HB formed by N36 R46E/A and C34 migrate faster than the wild-type 6-HB. This result suggests that the mutants can form 6-HB as wild-type NHR, contingent on a change in conformation, resulting in different migration rates. Additionally, N36 R46E/A-C34 complexes, especially the N36 R46E -C34 complex, displayed weaker fluorescence bands concomitant with a recovery of C34-FAM band, suggesting that the mutant complexes with residues having alternative charge and size are in a more unstable conformation, highlighting the important role that the R46 plays in forming the functional conformation of the 6-HB.

### Non-conservative Substitutions of R46 Impaired gp41-mediated HIV-1 Entry

**Figure 5:**
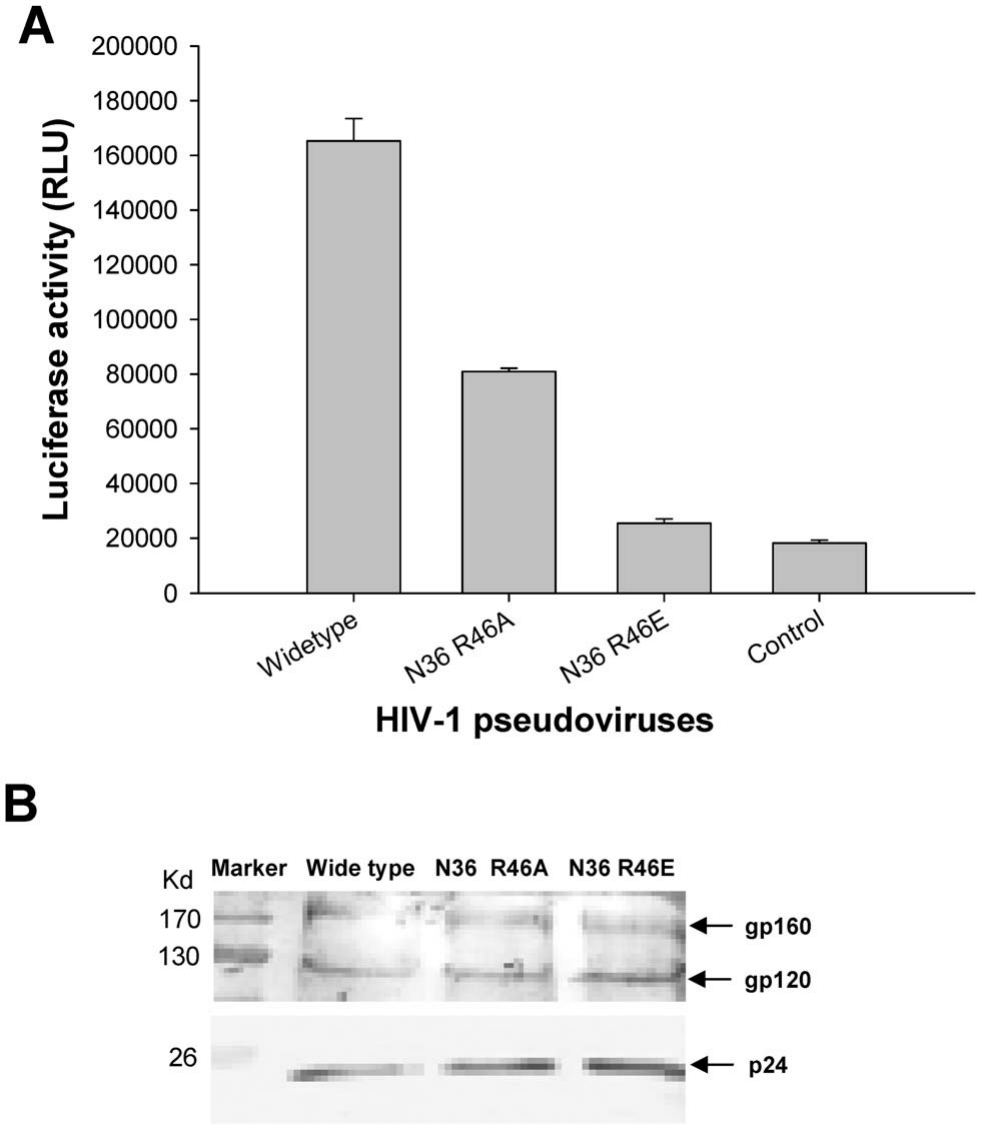
Effect of R46 mutations in gp41 NHR region on viral infectivity and Env expression. (**A**) Infectivity of HIV-1 pseudoviruses carrying wild-type N36 and its mutants. Luciferase assay was used to measure the single-cycle infection of pseudovirus on TZM-bl cells. (**B**) Expression of gp120 and gp160 on pseudoviruses bearing wild-type N36 sequence and its mutants as determined by Western blot.

The main function of the viral envelope is fusion with the target cell membrane to mediate viral entry. Thus, it is of great importance to observe the influence of the mutant residue on the entry of the virus. To probe the function of R46, we performed a luciferase-based single-cycle pseudovirus infection assay to analyze the effects of substitutions of R46 with A46 and E46, respectively. In this way, viral entry could be observed by luciferase activity. Compared with the wild type, the pseudoviruses with non-conservative substitution of R46A in gp41 exhibited reduced infectivity (**Figure 5A**). Consistent with our discovery, Sen et al. have also shown that the virus with R46A mutation exhibited much lower viral entry activity (only 37% of wild type) and impaired fusion activity, although the expression of gp120/gp41 did not significantly change [26]. In comparison, the pseudovirus with R46E almost lost the capacity to infect cells, while the pseudoviruses with wild type or mutant Env expressed similar level of gp160 and gp120 (**Figure 5B**). These results suggest that R46A and R46E mutations have no significant effect on the Env expression and proteolytic processing and that different kinds of mutations with correspondingly different electrical properties impair viral entry in varying degrees. This finding demonstrated that the R46 residue in NHR of gp41 is crucial for HIV-1 entry.

### Non-conserved Substitutions of R46 and E137 Resulted in Decreased Inhibitory Activity of the NHR Peptide N36 and CHR Peptide C34, Respectively, on HIV-1-Mediated Cell-to-Cell Fusion and HIV-1 Infection

Our results demonstrated that the non-conservative substitutions of R46 could affect the conformation and stability of the 6-HB formed by N36 and C34, implying that the mutants of R46 may affect 6-HB formation and the antiviral activity of the N- and C-peptides. We thus measured the inhibitory activity of N36 and C34 and their mutants on HIV-1_IIIB_-mediated cell-cell fusion and HIV-1_ IIIB_ infection. As shown in **Table 2**, the inhibitory potency of the mutant peptides N36 R46A and N36 R46E was significantly affected (P<0.01) with a reduction of about 6-fold relative to the potency of N36 in inhibiting HIV-1_IIIB_-mediated cell-cell fusion and 0.6- and 1.2-fold lower, respectively, than N36 in inhibiting HIV-1_ IIIB_ infection. Similarly, the mutant peptides C34 E137A and C34 E137R also exhibited lower potency than C34 in inhibiting the HIV-1_IIIB_-mediated cell-cell fusion and HIV-1_ IIIB_ infection. These results suggest that non-conserved substitutions of R46 and E137 affect their antiviral activity.

**Table 2:** Inhibition of the peptides N36, C34 and their mutants on HIV-1_IIIB_-mediated cell-cell fusion and HIV-1_ IIIB_ infection.

Peptides	IC_50_ (nM) for inhibition of			
	HIV-1_IIIB_–mediatedcell-cell fusion	*P* *	HIV-1_IIIB_infection	*P* *
N36	415.1±17.2		720.2±97.4	
N36 R46A	2860.4±545.6	0.0029	1151.6±121.1	0.0096
N36 R46E	2976.3±54.8	0.0000	1584.0±125.9	0.0010
C34	2.543±0.528		5.283±0.331	
C34 E137A	4.681±0.029	0.0039	12.155±1.276	0.0080
C34 E137R	5.318±1.289	0.0083	22.082±1.798	0.0030

* Compared with the corresponding wild-type peptide N36 or C34 (Student *t*-test was performed using Excel).

## Discussion

As a precondition of viral entry, three CHRs must pack in an antiparallel manner into the hydrophobic grooves of the NHR-trimer to form a fusion-active gp41 core structure [2], [3], [4]. Although hydrophobic interactions are the main driving force for this inter-helical packing, inter- and intra-helical hydrogen bonds distributed along the hydrophobic contacts also contribute to stabilization of the packing, which ultimately leads to the virus-cell fusion. In our study, the conformation and stability of fusion-active six-helical bundle modeled by mutant N36 and C34 were remarkably impaired. Noticeably, R46E substitution resulted in the most dramatic change, as observed in the CD spectra **(** **Figure 3**), in line with reduced thermal stability, binding affinity tested by FN-PAGE, and the lowest N36-mediated antiviral activity, as well as impairing Env-mediated viral entry, whereas R46A had relatively modest effect. In comparison, mutation of E137 only slightly changed the secondary structure of the 6-HB, indicating that the salt bridge formed by R46 and E137 is less important, as a stabilization factor, than the hydrogen-bond network around R46 in terms of 6-HB formation. Collectively, R46 is a novel and key determinant of viral entry.

Recently, it has been shown that the replacements of certain non-conserved solvent-exposed residues out of the cavity-forming region could abolish viral entry as dramatically as that observed by replacing the conserved and buried residues [26]. Generally, in the wild-type isolates, most of the residues which impair the critical functions tend to be relatively conserved. These critical residues were located in or around a novel motif both in the NHR and CHR, termed “glutamine layer” [21], [25], [31]. Few studies have emphasized the impact of point mutations in this region, and little is known about the functional effect of these mutations [13], [21]. The two “glutamine layers,” which appeared to be involved in the inter- and intra-helical contacts, may have a remarkable effect on the membrane fusion of host cell and virus through rearrangement of hydrogen bonds. Thus, we hypothesized that Arg46, an exposed, moderately variable residue around this glutamine-rich region, might confer a significant strength to the inter- or intra-helical interactions and thus impact the formation and stabilization of the 6-HB (**Figure 1C**). As shown in **Table 1**, R46 is involved in the hydrogen-bond network, and mutations of R46 can induce a rearrangement of this network involving more adjacent residues. Thus, we conclude that R46A and R46E mutations can impair the stability of gp41 by partially destroying the hydrogen-bond network.

Since escape mutants result from the clinical use of anti-HIV drugs, identification and validation of novel molecular targets for developing new antiviral therapeutics with improved efficacy and resistance profile have become of paramount importance. As presented in this work, hydrogen bonds have a significant effect on the stability of gp41. Thus, shedding light on the hydrogen-bond network within the “glutamine layer” may provide useful information for identification of such novel drug targets. Since the residues at the positions “*b*” and “*f*” in the α-helix of gp41 are located at the hydrophilic surface of the gp41 N-trimer and 6-HB, these residues can form the epitopes which are accessible to antibodies, even after the formation of fusion intermediate [5], [27]. Therefore, changes of these residues may significantly affect the conformation of the epitopes and the immunogenicity or antigenicity.

In conclusion, the hydrogen-bond network within 6-HB may serve as a new target of HIV fusion inhibition or as an attractive antigenic determinant for eliciting anti-HIV immune responses. Therefore, characterization of the role of such key residues as Agr46 located near or within the “glutamine layer” may provide important information for understanding the mechanism of gp41-mediated viral fusion and designing novel HIV fusion inhibitors as AIDS therapeutics or immunogens as AIDS vaccine candidates.

## Materials and Methods

### Prediction of Possible Interaction in 6-HB

The possible salt bridges and main hydrogen bonds in 6-HB were predicted by using MOE software (Chemical Computing Group, Inc., Montreal, Quebec, Canada). In brief, 1AIK was downloaded from the Protein Data Bank and analyzed with the MOE program. The information of the hydrogen bonds with energy over 0.2 Kcal/mol in 6-HB were collected. The structure energy of 6-HB which contained mutated residues was minimized by the MOE program and copied.

### Synthesis of Wild-type and Mutant Peptides

As shown in **Figure 1**A, peptides N36 (residues 546–581, SGIVQQQNNLLRAIEAQQHLLQLTVWGIKQLQ ARIL) and C34 (residues 628–661, WMEWDREINNYTSLIHSLIEESQNQQEKNEQELL), as well as their mutants (N36 R46A, N36 R46E, C34 E137A, and C34 E137R), were synthesized using the FMOC method. To purify and identify the synthesized peptides to homogeneity (>95% purity), we performed high-performance liquid chromatography (HPLC) and laser desorption mass spectrometry (PerSeptive Biosystems, Framingham, MA). Then by UV absorbance, together with a theoretically calculated molar extinction coefficient ε (280 nm) of 5500 mol/L^–1^.cm^–1^ and 1490 mol/L^–1^.cm^–1^ based on the number of tryptophan (Trp) residues and tyrosine (Tyr) residues (all peptides tested contain Trp and/or Tyr), respectively [32], the concentration of peptides was subsequently measured. Prior to CD, dialyzation of all peptides against PBS was required.

### Circular Dichroism (CD) Spectroscopic Analysis

CD measurements were applied to determine the helicity as formerly described [33], [34]. In brief, we dissolved C34 and N36 and their mutants in phosphate buffer solution (PBS), pH 7.2. Each 8 µM peptides or 8 µM mixtures of each peptide in PBS were incubated at 37°C for 30 min. We obtained the CD spectrum of each sample on a Jasco spectropolarimeter (Model J-715, Jasco Inc., Japan) at 20°C with a 5 nm bandwidth, 0.5 nm resolution, 0.1 cm path length, and an average time of 5.0 sec. The subtraction of a blank corresponding to the solvent composition of each sample was used to correct the spectra. Peptide interactions were analyzed based on Lawless’s protocol via a comparison between the spectrum of the peptide mixture (experimental spectrum) and the sum of the individual spectra of the peptides at the identical concentration and experimental condition (calculated noninteracting spectrum). The percent of alpha–helix was calculated by an online K2D program (http://www.embl.de/~andrade/k2d.html). To determine the reversibility, we performed thermal denaturation where a thermal gradient of 2°C/min from 20°C to 100°C was applied. Subsequently, the peptides were cooled to 20°C for 30 min and then again heated to 100°C. We monitored all the denaturation procedures at the 222 nm wavelength. After smoothening the melting curve, we applied Jasco software to analyze the thermal unfolding transition (*Tm*) value.

### HIV-1-mediated Cell–cell Fusion

To detect HIV-1 mediated cell-cell fusion, we performed a dye transfer assay as previously described [6]. Briefly, we incubated calcein-AM-labeled H9/HIV-1_IIIB_-infected cells with MT-2 cells (ratio = 1∶5) at 37°C for 2 h in the presence of N36 or its mutants (both H9/HIV-1_IIIB_-infected cells and MT-2 cells were obtained from the NIH AIDS Research and Reference Reagent Program). Then under an inverted fluorescence microscope (Zeiss, Germany) with an eyepiece micrometer disc, we calculated the fused and unfused calcein-labeled HIV-1-infected cells. Previously developed methods were applied to calculate the percent inhibition of cell-cell fusion and the IC50 values [35].

### Inhibition of Peptides on HIV-1_IIIB_ Infection

The inhibitory activity of the NHR- and CHR-peptides on HIV-1_IIIB_ infection was determined as previously described [36]. In brief, 1×10^4^ MT-2 cells were infected with HIV-1_ IIIB_ at 100 TCID_50_ (50% tissue culture infective dose) in 200 µl culture medium in the presence or absence of the test peptide overnight. Then the culture supernatants were removed, and fresh media were added. On the fourth day post-infection, 100 µl of culture supernatants were collected from each well, mixed with equal volumes of 5% Triton X-100 and assayed for p24 antigen by an in-house ELISA as described before [36].

### Fluorescence N-PAGE (FN-PAGE) for Detecting 6-HB Formation

As previously described, we determined the role of N36 mutants played in gp41 6-HB formation with C34-FAM by N-PAGE using 18% precast Tris-Glycine gels (Invitrogen). Then we imaged the fluorescence bands in the gel by the FluorChem 8800 Imaging System using a transillumination UV light source with excitation wavelength at 480 nm and a fluorescence filter with emission wavelength at 525 nm. The gel was restained with Coomassie Blue [34], and the second image was taken with a FluorChem 8800 imaging system using white light.

### Construction of HIV-1 Env Mutants

To this research, we applied the plasmid encoding HIV-1 HXB2-Env provided by Dr. Kathleen Page and Dr. Dan Littman through the NIH AIDS Research and Reference Reagent Program. Two HXB2-Env mutants (R46A and R46E) were constructed by site-directed mutagenesis using the QuickChange XL kit (Strategene, La Jolla, CA) and confirmed by DNA sequencing. To construct HIV-1 Env mutants, we designed and synthesized the primers on the basis of the manufacturer’s instructions.

### Generation of HIV-1 Pseudovirus

HIV-1 pseudovirus was created according to previously described methods [35], [37]. HEK293T cells (purchased from ATCC, Manassas, VA) were co-transfected with two plasmids that encode wild type (WT) or mutant HXB2-Env and Env-defective, luciferase expressing HIV-1 genome (pNL4-3.luc.R^–^E^–^), respectively, using Fugene 6 reagents (Boehringer-Mannheim, Indianapolis, IN). The supernatants containing HIV-1 pseudovirus were harvested 48 h post-transfection.

### Single-cycle Infection Assay

In short, U87-T4-CXCR4 cells (obtained from the NIH AIDS Research and Reference Reagent Program) were plated at 2×10^4^cells/well in 96-well tissue culture plates and grown overnight. The cells were incubated overnight with the supernatants containing pseudovirus. An additional 48 h was required to refeed and hatch the culture. Cells were washed by phosphate-buffered saline (PBS) prior to lysing by lysis reagent included in a luciferase kit (Promega, Madison, WI). Aliquots of cell lysates were transferred to 96-well Costar flat-bottom luminometer plates (Corning Costar, Corning, NY), followed by addition of the luciferase substrate (Promega) to plate. Immediately, we determined the relative light units (RLU) using an Ultra 384 luminometer (Tecan US).

### Analysis of Viral Env Expression

Viral Env express was analyzed as previously procedure [16]. Briefly, 293T cells were co-transfected with plasmid coding the Env of HXB2 (WT) or its mutants (R46A or R46E in NHR of gp41) and the plasmid pNL_4-3._LucR-E-. The medium was changed 12 h post-transfection. supernatants containing the pseudoviruses were collected 48 h post-transfection and concentrated by centrifugation at 20,000 g for 3 hours. Supernatants were discarded and the virus pellets were resuspended with loading buffer. After the resolved in 12% SDS-PAGE, the proteins were transferred onto PDVF membranes. The blots were blocked with Tris buffered saline containing 5% fat free dry milk and 0.1% Tween-20 and probed by sheep-anti-HIV-1 gp120 (AALTOCorp.), anti-sheep IgG-biotin and anti-biotin-alkaline phosphatase, sequentially. P24, as a control, was probed by rabbit-anti-p24 (in-house antibody) and anti-rabbit IgG-alkaline phosphatase, sequentially.

## References

[1] Freed EO, Martin MA (1995) The role of human-immunodeficiency virus type-1 envelope glycoproeins in viurs-inection. J Biol Chem 270: 23883–23886. 7592573 10.1074/jbc.270.41.23883

[2] Eckert DM, Kim PS (2001) Mechanisms of viral membrane fusion and its inhibition. Annu Rev Biochem 70: 777–810. 11395423 10.1146/annurev.biochem.70.1.777

[3] Esser MT, Mori T, Mondor I, Sattentau QJ, Dey B, et al (1999) Cyanovirin-N binds to gp120 to interfere with CD4-dependent human immunodeficiency virus type 1 virion binding, fusion, and infectivity but does not affect the CD4 binding site on gp120 or soluble CD4-induced conformational changes in gp120. J Virol 73: 4360–4371. 10196334 10.1128/jvi.73.5.4360-4371.1999 PMC104217

[4] Wyatt R, Sodroski J (1998) The HIV-1 envelope glycoproteins: Fusogens, antigens, and immunogens. Science 280: 1884–1888. 9632381 10.1126/science.280.5371.1884

[5] Chan DC, Fass D, Berger JM, Kim PS (1997) Core structure of gp41 from the HIV envelope glycoprotein. Cell 89: 263–273. 9108481 10.1016/s0092-8674(00)80205-6

[6] Jiang S, Lin K, Strick N, Neurath AR (1993) HIV-1 inhibition by a peptide. Nature 365: 113. 8371754 10.1038/365113a0

[7] Chan DC, Kim PS (1998) HIV entry and its inhibition. Cell 93: 681–684. 9630213 10.1016/s0092-8674(00)81430-0

[8] Dimitrov DS (1997) How do viruses enter cells? The HIV coreceptors teach us a lesson of complexity. Cell 91: 721–730. 9413981 10.1016/s0092-8674(00)80460-2

[9] Feng Y, Broder CC, Kennedy PE, Berger EA (1996) HIV-1 entry cofactor: Functional cDNA cloning of a seven-transmembrane, G protein-coupled receptor. Science 272: 872–877. 8629022 10.1126/science.272.5263.872

[10] Bianchi E, Finotto M, Ingallinella P, Hrin R, Carella AV, et al (2005) Covalent stabilization of coiled coils of the HIV gp41 N region yields extremely potent and broad inhibitors of viral infection. Proc Natl Acad Sci U S A 102: 12903–12908. 16129831 10.1073/pnas.0502449102 PMC1200264

[11] Gochin M, Cai L (2009) The role of amphiphilicity and negative charge in glycoprotein 41 interactions in the hydrophobic pocket. J Med Chem 52: 4338–4344. 19534533 10.1021/jm900190q PMC2724993

[12] Follis KE, Larson SJ, Lu M, Nunberg JH (2002) Genetic evidence that interhelical packing interactions in the gp41 core are critical for transition of the human immunodeficiency virus type 1 envelope glycoprotein to the fusion-active state. J Virol 76: 7356–7362. 12072535 10.1128/JVI.76.14.7356-7362.2002 PMC136323

[13] Cao J, Bergeron L, Helseth E, Thali M, Repke H, et al (1993) Effects of amino acid changes in the extracellular domain of the human immunodeficiency virus type 1 gp41 envelope glycoprotein. J Virol 67: 2747–2755. 8474172 10.1128/jvi.67.5.2747-2755.1993 PMC237598

[14] Lu M, Stoller MO, Wang S, Liu J, Fagan MB, et al (2001) Structural and functional analysis of interhelical interactions in the human immunodeficiency virus type 1 gp41 envelope glycoprotein by alanine-scanning mutagenesis. J Virol 75: 11146–11156. 11602754 10.1128/JVI.75.22.11146-11156.2001 PMC114694

[15] Weng Y, Yang Z, Weiss CD (2000) Structure-function studies of the self-assembly domain of the human immunodeficiency virus type 1 transmembrane protein gp41. J Virol 74: 5368–5372. 10799616 10.1128/jvi.74.11.5368-5372.2000 PMC110894

[16] Weng Y, Weiss CD (1998) Mutational analysis of residues in the coiled-coil domain of human immunodeficiency virus type 1 transmembrane protein gp41. J Virol 72: 9676–9682. 9811701 10.1128/jvi.72.12.9676-9682.1998 PMC110477

[17] Wang S, York J, Shu W, Stoller MO, Nunberg JH, et al (2002) Interhelical interactions in the gp41 core: implications for activation of HIV-1 membrane fusion. Biochemistry 41: 7283–7292. 12044159 10.1021/bi025648y

[18] Ji H, Shu W, Burling FT, Jiang S, Lu M (1999) Inhibition of human immunodeficiency virus type 1 infectivity by the gp41 core: role of a conserved hydrophobic cavity in membrane fusion. J Virol 73: 8578–8586. 10482611 10.1128/jvi.73.10.8578-8586.1999 PMC112878

[19] Lalezari JP, Henry K, O’Hearn M, Montaner JS, Piliero PJ, et al (2003) Enfuvirtide, an HIV-1 fusion inhibitor, for drug-resistant HIV infection in North and South America. N Engl J Med 348: 2175–2185. 12637625 10.1056/NEJMoa035026

[20] Dai SJ, Dou GF, Qiang XH, Song HF, Tang ZM, et al (2005) Pharmacokinetics of sifuvirtide, a novel anti-HIV-1 peptide, in monkeys and its inhibitory concentration in vitro. Acta Pharmacol Sin 26: 1274–1280. 16174446 10.1111/j.1745-7254.2005.00163.x

[21] Suntoke TR, Chan DC (2005) The fusion activity of HIV-1 gp41 depends on interhelical interactions. J Biol Chem 280: 19852–19857. 15772068 10.1074/jbc.M502196200

[22] Shu W, Liu J, Ji H, Radigen L, Jiang S, et al (2000) Helical interactions in the HIV-1 gp41 core reveal structural basis for the inhibitory activity of gp41 peptides. Biochemistry 39: 1634–1642. 10677212 10.1021/bi9921687

[23] Tan K, Liu J, Wang J, Shen S, Lu M (1997) Atomic structure of a thermostable subdomain of HIV-1 gp41. Proc Natl Acad Sci U S A 94: 12303–12308. 9356444 10.1073/pnas.94.23.12303 PMC24915

[24] Weissenhorn W, Calder LJ, Dessen A, Laue T, Skehel JJ, et al (1997) Assembly of a rod-shaped chimera of a trimeric GCN4 zipper and the HIV-1 gp41 ectodomain expressed in Escherichia coli. Proc Natl Acad Sci U S A 94: 6065–6069. 9177169 10.1073/pnas.94.12.6065 PMC21001

[25] Malashkevich VN, Chan DC, Chutkowski CT, Kim PS (1998) Crystal structure of the simian immunodeficiency virus (SIV) gp41 core: conserved helical interactions underlie the broad inhibitory activity of gp41 peptides. Proc Natl Acad Sci U S A 95: 9134–9139. 9689046 10.1073/pnas.95.16.9134 PMC21304

[26] Sen J, Yan T, Wang J, Rong L, Tao L, et al (2010) Alanine scanning mutagenesis of HIV-1 gp41 heptad repeat 1: insight into the gp120-gp41 interaction. Biochemistry 49: 5057–5065. 20481578 10.1021/bi1005267

[27] Liu J, Shu W, Fagan MB, Nunberg JH, Lu M (2001) Structural and functional analysis of the HIV gp41 core containing an Ile573 to Thr substitution: Implications for membrane fusion. Biochemistry 40: 2797–2807. 11258890 10.1021/bi0024759

[28] Chang DK, Cheng SF, Yang SH (2000) A helix initiation motif, XLLRA, is stabilized by hydrogen bond, hydrophobic and van der Waals interactions. Biochim Biophys Acta 1478: 39–50. 10719173 10.1016/s0167-4838(99)00286-1

[29] MOE (2010) Chemical Computing Group, Inc. Montreal, Quebec, Canada version 2010.

[30] He Y, Liu S, Jing W, Lu H, Cai D, et al (2007) Conserved residue Lys574 in the cavity of HIV-1 Gp41 coiled-coil domain is critical for six-helix bundle stability and virus entry. J Biol Chem 282: 25631–25639. 17616522 10.1074/jbc.M703781200

[31] Weissenhorn W, Dessen A, Harrison SC, Skehel JJ, Wiley DC (1997) Atomic structure of the ectodomain from HIV-1 gp41. Nature 387: 426–430. 9163431 10.1038/387426a0

[32] Edelhoch H (1967) Spectrocopic determination of tryptophan and tryosine in proteins. Biochemistry 6: 1948–1954. 6049437 10.1021/bi00859a010

[33] Liu S, Lu H, Niu J, Xu Y, Wu S, et al (2005) Different from the HIV fusion inhibitor C34, the anti-HIV drug Fuzeon (T-20) inhibits HIV-1 entry by targeting multiple sites in gp41 and gp120. J Biol Chem 280: 11259–11273. 15640162 10.1074/jbc.M411141200

[34] Liu S, Jing W, Cheung B, Lu H, Sun J, et al (2007) HIV gp41 C-terminal heptad repeat contains multifunctional domains. Relation to mechanisms of action of anti-HIV peptides. J Biol Chem 282: 9612–9620. 17276993 10.1074/jbc.M609148200

[35] Jiang S, Lu H, Liu S, Zhao Q, He Y, et al (2004) N-substituted pyrrole derivatives as novel human immunodeficiency virus type 1 entry inhibitors that interfere with the gp41 six-helix bundle formation and block virus fusion. Antimicrob Agents Chemother 48: 4349–4359. 15504864 10.1128/AAC.48.11.4349-4359.2004 PMC525433

[36] Pan C, Cai L, Lu H, Qi Z, Jiang S (2009) Combinations of the first and next generations of human immunodeficiency virus (HIV) fusion inhibitors exhibit a highly potent synergistic effect against enfuvirtide- sensitive and -resistant HIV type 1 strains. J Virol 83: 7862–7872. 19493996 10.1128/JVI.00168-09 PMC2715752

[37] He Y, D’Agostino P, Pinter A (2003) Analysis of the immunogenic properties of a single-chain polypeptide analogue of the HIV-1 gp120-CD4 complex in transgenic mice that produce human immunoglobulins. Vaccine 21: 4421–4429. 14505925 10.1016/s0264-410x(03)00451-1

